# Tapencarium (RZL-012) for Flank Fat Reduction: A Proof-of-Concept Study

**DOI:** 10.1093/asjof/ojad094

**Published:** 2023-10-27

**Authors:** Sachin M Shridharani, MacKenzie L Kennedy, Racheli Gueta, Patricia Walker

## Abstract

**Background:**

RZL-012 is a novel cytolytic drug that has shown promise in reducing localized fat deposits in a single treatment session.

**Objectives:**

To assess the safety and efficacy of injecting RZL-012 to the flanks.

**Methods:**

A double-blind, placebo-controlled, proof of concept study randomized 12 patients to receive RZL-012 injections in 1 flank and placebo injections in the contralateral flank. After 12 weeks of follow-up, patients could receive RZL-012 in the placebo-treated flank and undergo follow-up for 12 weeks in the open-label phase.

**Results:**

At 12 weeks, Investigator Global Aesthetic Improvement Scale assessments showed improvement for 90.9% of RZL-012-treated flanks and 0% of placebo-treated flanks (*P* < .0001), 81.8% of patients were satisfied with the RZL-012-treated flanks, and 9.1% were satisfied with the placebo-treated flanks (*P* = .0019). Volume reduction measured on 3-dimensional images was a mean 37.27 mL, which was significantly greater than placebo (*P* = .0052). The product was well tolerated, with no clinically significant trends in laboratory values, electrocardiograms, or vital signs. Pharmacokinetic analyses demonstrated that RZL-012 is quickly absorbed, reaches maximum concentration in approximately 1.67 h, and has a half-life of 9.1 h. The mean maximal concentration of RZL-012 found in the blood was <1 µg/mL.

**Conclusions:**

RZL-012 is a promising option for injectable fat reduction of the flanks in a single treatment session. The drug was well tolerated in this small patient population, with no concerning safety signals, and it had indications of efficacy. Further research is needed in large Phase 2 studies with robust efficacy measurements to confirm these early findings.

**Level of Evidence: 2:**

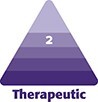

Body contouring procedures are becoming increasingly common to address unwanted localized fat deposits, with noninvasive fat reduction procedures surpassing liposuction in frequency in the United States. Liposuction procedures saw a 40% decrease from 2000 to 2020, whereas noninvasive fat reduction procedures have been on the increase.^1^ Currently available devices include those employing cryolipolysis, lasers, radiofrequency, focused ultrasound, and high-intensity focused electromagnetic technologies, although these generally require multiple treatments at various intervals.^2^ For example, cryolipolysis treatment of the flanks may need 4 to 6 treatment cycles spaced 4 to 8 weeks apart to obtain a visible clinical outcome.^3^ The flanks were the second most common area to be treated in a survey of people undergoing cryolipolysis for the first time, and the primary reasons given for seeking the procedure were to correct an aesthetic imperfection without surgery and to feel more self-confident.^4^

To address localized fat deposits in a single treatment session, Raziel Therapeutics developed a novel cytolytic drug, tapencarium (RZL-012, 5-(3,6-dibromo-9H-carbazol-9-yl)-*N*,*N*,*N*-trimethylpentan-1-aminium chloride), which has shown promise in reducing fat deposits in the submental area and in patients with Dercum's disease.^5,6^ Injection of RZL-012 into fat tissue disrupts cell membrane integrity, subsequently killing fat cells, after which the treated area goes through the normal inflammatory and wound-healing process, with complete clearance of dead fat tissue seen at 84 days after injection in animal studies.^7^

In the submental fat Phase 2b dose-ranging study, the mean dose of RZL-012 utilized in the high-dose group was 244 mg/patient.^6^ Because the flanks are a larger treatment area than the submental area, a larger dose of RZL-012 will be needed, so we undertook a small proof-of-concept study to assess the safety and efficacy of injecting 412.5 mg of RZL-012 to the flank in a single treatment session.

## METHODS

### Study Design

A single investigational site conducted this Phase 2 proof-of-concept study to evaluate the safety and efficacy of RZL-012 in patients seeking fat reduction in the flanks. In the initial double-blind, placebo-controlled phase of the study, patients were randomized to receive RZL-012 injections in 1 flank and placebo injections in the contralateral flank. The single treatment session was followed by clinic visits at Weeks 1, 4, 8, and 12. Patients were then eligible to receive RZL-012 treatment in the flank that had received placebo treatment and enter the open-label phase of the study with the same follow-up visit schedule as the double-blind phase. Additional visits on the baseline day of initial treatment and the following day allowed for blood draws for half of the patients to characterize the pharmacokinetic profile of RZL-012.

The study was reviewed and approved by Advarra Institutional Review Board and was registered at www.clinicaltrials.gov (NCT05445557). Written consent was provided, by which the patients agreed to the use and analysis of their data. The study was conducted in 2022 from February to October.

### Patients

Key inclusion criteria were ages 18 to 65, body mass index of 22 to >30, a clearly visible and palpable flank fat, and a bilateral symmetrical flank appearance. Key exclusion criteria were any noninvasive fat reduction or invasive body contouring procedures in the flanks within 12 months, chronic systemic steroids or immunosuppressive drugs within 3 months, anticoagulation therapies within 1 week, or an abnormal coagulation profile.

### Treatment

**Figure 1.**
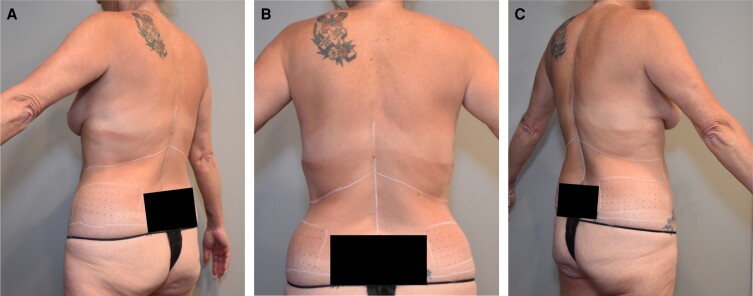
The photographs show a 55-year-old female patient with the marked tattoo grid on the (A) left and (B) from the back view, and (C) (B) right flanks.

Prior to injections, a temporary tattoo grid measuring 15 cm by 6 cm was placed on the bilateral flanks of each patient and delineated 1.5 cm between each injection point (Figure 1). Using a 27-gauge 1 inch needle pointing 90° perpendicular to the skin surface, 55 injections of 0.15 mL of active or placebo sterile solution were made to the fat layer of each flank for a total dose of 8.25 mL. The RZL-012 solution was 50 mg/mL, which equated to 7.5 mg per injection and a total of 412.5 mg for the flank. An ice pack was placed on the injected area for pain relief immediately afterward, and patients remained in injection position for 10 min after dosing.

### Assessments

At Weeks 4, 8, and 12, the investigator assessed the improvement of each flank on the 7-point Global Aesthetic Improvement Scale (GAIS; Table 1), and patients completed a satisfaction questionnaire by providing yes/no responses to 3 questions: (1) “for the right/left flank, are you satisfied with the treatment?,” (2) “would you have the treatment again in the right/left flank?,” and (3) “would you recommend the treatment to a friend?” Three-dimensional photographic images were captured at screening and all posttreatment visits and were used to calculate volume change via Canfield Vectra technology after treatment.

**Table 1.** Global Aesthetic Improvement Scale

Grade		Description
0	Completely clear	No evidence of fat; 100% improvement
1	Almost clear	Very significant clearance (≥90% to <100%); only traces remain
2	Marked improvement	Significant improvement (≥75% to <90%); some evidence of fat remains
3	Moderate improvement	Intermediate between slight and marked improvement (≥50% to <75%)
4	Slight improvement	Some improvement (≥25% to <50%); significance evidence of fat remains
5	No change	Fat has not changed from baseline condition (±25%)
6	Worse	Fat is worse than at baseline evaluation by ≥25% or more

Adverse events (AEs) were captured at all posttreatment visits, with particular attention to the treatment areas, and the investigator assessed all AEs for relationship to treatment and severity (mild, moderate, severe, life-threatening or disabling, death). At screening and Weeks 1, 4, and 12 in each study phase, electrocardiograms were performed, and blood samples were collected for assessment of hematology and serum chemistry. Vital signs (systolic and diastolic blood pressure in sitting position, pulse rate, respiratory rate, and body oral temperature) were collected at all study visits, including both before and after injections on study treatment days. Six patients additionally had blood samples drawn for pharmacokinetics predose, 30 min, 60 min, 2 h, 3 h, 4 h, 6 h, 8 h, 24 h, and 30 h after the initial injection session.

### Statistics

To compare results for the treated and control flank groups, the Mantel–Haenszel χ^2^ test was used for the GAIS, Fisher's exact test was used for patient satisfaction, and a paired *t* test was used for 3-dimensional volume change measurements. AEs were classified by MedDRA system organ class and preferred term, and all safety variables were summarized with descriptive statistics.

## RESULTS

### Patient Characteristics

Of the 12 patients enrolled, 10 were women and 2 were men. Ten patients were white, and 2 were African-American. Ethnicity was Hispanic or Latino for 2 patients. Mean age was 43 years, with a range of 26 to 61 years. All 12 patients were randomized and treated with RZL-012 in the left or right flank and placebo solution in the contralateral flank. Follow-up was achieved through 8 weeks for all patients and through 12 weeks for 11 patients. After completion of the double-blind phase of the study, 9 patients consented to enroll in the open-label phase and were injected with RZL-012 in the flank that had previously been treated with placebo. For the 2 patients who declined contralateral flank treatment, 1 was due to a perceived lack of efficacy, and 1 was due to painfulness of treatment. All 9 patients completed the 12-week open-label follow-up period. All injected flanks in both study phases received 55 injections with a total injected volume of 8.25 mL.

### Safety

Because this was the first study to encompass a RZL-012 dose of 412.5 mg in a single treatment session, the primary study endpoint was evaluation of safety after a single treatment via AEs, skin irritancy, laboratory tests, and electrocardiograms. In the double-blind phase of the study, there were 56 AEs in RZL-012-treated flanks and 47 in placebo-treated flanks (Table 2). All skin irritancy AEs were of mild severity, with pruritus occurring more frequently in the RZL-012 flanks and lasting longer (mean of 42 days vs 15 days). Erythema and ecchymosis occurred with similar frequency and duration for both groups. Other AEs with greater incidence and longer duration in the RZL-012 flanks were hypoaesthesia (28 days vs 11 days) and tenderness (45 days vs 2 days). There were no reports of persistent firmness/induration. The AE profile was similar in the open-label study phase, except for 3 flanks exhibiting transient hyperpigmentation (mean duration of 100 days). In both study phases, there were no AEs that were rated severe or considered to be serious. No clinically significant trends were related to treatment for hematology and chemistry laboratory values, electrocardiograms, or vital signs in either study phase.

**Table 2.** Adverse Events by Severity in Double-Blind Study Phase

MedDRA system organ class/preferred term/severity			RZL-012 (*n* = 12)		Placebo (*n* = 12)	
			*n*	%	*n*	%
General disorders and administration site conditions	Edema	Mild	0	0	8	66.7
		Moderate	11	91.7	4	33.3
	Pain	Mild	2	16.7	6	50.0
		Moderate	10	83.3	6	50.0
	Tenderness	Mild	4	33.3	1	8.3
Skin and subcutaneous tissue disorders	Pruritus	Mild	11	91.7	7	58.3
	Erythema	Mild	7	58.3	6	50.0
	Ecchymosis	Mild	6	50.0	8	66.7
Nervous system disorders	Hypoaesthesia	Mild	5	41.7	1	8.3

### Efficacy

**Figure 2.**
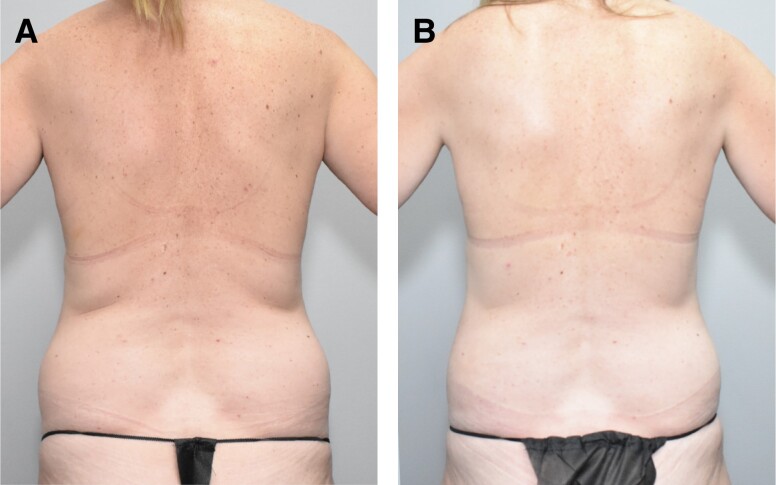
(A) Before and (B) after photographs showing a 48-year-old female patient's baseline and Week 12 images. The left flank was treated with RZL-012, and GAIS was rated as marked improvement.

**Figure 3.**
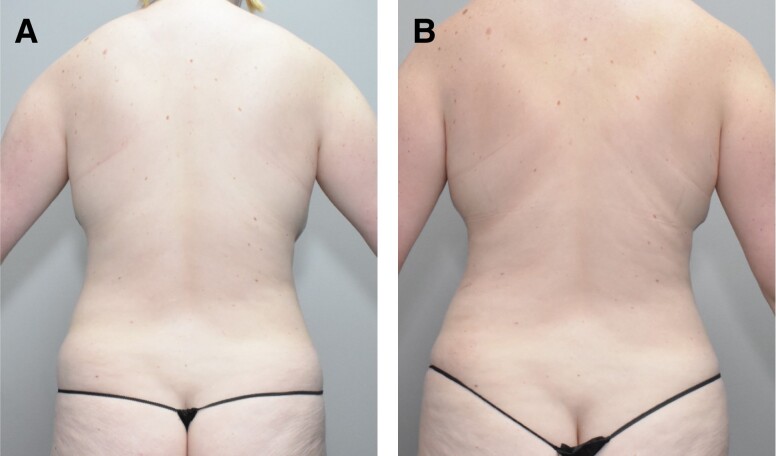
(A) Before and (B) after photographs show a 28-year-old female patient's baseline and Week 24 images following treatment with RZL-012 on the right flank in the blinded phase and the left flank in the open-label phase.

Secondary endpoints included a comparison of GAIS, patient satisfaction, and volume change measurements between the 2 treatment groups in the double-blind phase. Investigator GAIS assessments at 12 weeks after initial treatment showed improvement for 90.9% of RZL-012-treated flanks (63.6% marked improvement, 18.2% moderate improvement, and 9.1% slight improvement) and 0% of placebo-treated flanks (*P* < .0001). In the open-label study phase, 100% of treated flanks were improved on the GAIS (22.2% moderate improvement and 77.8% slight improvement). Photographic examples of baseline and Week 12 in the double-blind phase are provided in Figures 2 and 3. Of note, clinically meaningful 3-dimensional changes in volume and contouring can be difficult to visualize in photographs.

At 12 weeks after initial treatment, 81.8% of patients were satisfied with the RZL-012-treated flanks and 9.1% with the placebo-treated flanks (*P* = .0019). Two-thirds of patients (66.7%) were satisfied with treatment at 12 weeks in the open-label study phase. Additionally, at 12 weeks after initial treatment, 63.6% of patients reported that they would have the treatment again in the RZL-012-treated flank, and 81.8% would recommend RZL-012 to a friend. Flank volume reduction as measured on 3-dimensional images using Canfield Vectra technology was a mean of 37.27 mL at 12 weeks after initial treatment with RZL-012, which was significantly greater than placebo (*P* = .0052). In the open-label phase, volume reduction was a mean 48.85 mL at 12 weeks.

### Pharmacokinetics

Characterization of the pharmacokinetic profile of RZL-012 was also a secondary endpoint of the study. An analysis of blood samples from 6 patients demonstrated that RZL-012 is quickly absorbed, reaches maximum concentration in approximately 1.67 h, and has a half-life of 9.1 h. The mean maximal concentration of RZL-012 found in the blood was <1 µg/mL.

## DISCUSSION

The higher dosage of RZL-012 used for the flanks in this study compared with the earlier submental fat study did not show any concerning safety signals either for the single treatment session or for a second treatment in patients who participated in the open-label study phase. RZL-012 was safe and well tolerated based on AEs, laboratory parameters, electrocardiograms, and vital signs in this proof-of-concept study.

Efficacy results, although in a small population, were also encouraging, with RZL-012-treated flanks faring significantly better than placebo-treated flanks on the GAIS, patient satisfaction, and volume reduction measurements on 3-dimensional images. However, these endpoints are not robust and would not support a Phase 3 study. In contrast, the RZL-012 submental fat Phase 2b study employed validated clinician- and patient-reported outcome scales and magnetic resonance imaging (MRI) for primary and secondary endpoints.^6^

Studies of devices for body fat reduction have primarily used efficacy endpoints that are subjective, such as correct identification of pretreatment or posttreatment photographs, or unreliable, such as circumference or caliper measurements that are imprecise, or ultrasound imaging that is affected by pressure on the transducer. Circumference, caliper, and ultrasound measurements are all operator dependent, and, thus, unreliable. Three-dimensional imaging measurements bring more objectivity but are still not immune to operator error or a lack of standardization across anatomic landmarks.^8^

Recent studies have utilized MRI to demonstrate fat reduction following high-intensity focused electromagnetic treatments alone or in combination with radiofrequency,^9-13^ and MRI has also been used to measure submental fat reduction following deoxycholic acid treatments.^14^ Other objective measurements proposed for assessing reduction of adipose tissue are high-resolution ultrasound and diffuse optical spectroscopic imaging, the latter of which was tested in a patient undergoing cryolipolysis, in whom a decrease in the adipose layer was shown 60 days after treatment.^15,16^ All of these are potential efficacy measures that could be explored for future phase 2 dose-ranging studies. Because this was a proof-of-concept study, it has many limitations, including a small sample size, a single treatment session, and a lack of robust efficacy endpoints.

## CONCLUSIONS

Based on this proof-of-concept study, RZL-012 is a promising option for injectable fat reduction of the flanks in a single treatment session. The drug was well tolerated in this small patient population with no concerning safety signals and had indications of efficacy. Further research is needed in large Phase 2 studies with robust efficacy measurements to confirm these early findings. Endpoint development and study design are under way as next steps forward in developing RZL-012 for use in large body areas.

## References

[1] American Society of Plastic Surgeons . Plastic Surgery Statistics Report 2020. Accessed August 2, 2023. https://www.plasticsurgery.org/documents/News/Statistics/2020/plastic-surgery-statistics-full-report-2020.pdf

[2] Murgia RD , Noell C, Weiss M, Weiss R. Body contouring for fat and muscle in aesthetics: review and debate. Clin Dermatol. 2022;40(1):29–34. doi: 10.1016/j.clindermatol.2021.08.009 35190061

[3] Altmann J , Burns AJ, Kilmer SL, et al Global expert opinion on cryolipolysis treatment recommendations and considerations: a modified Delphi study. Aesthet Surg J Open Forum. 2022;4:ojac008. doi: 10.1093/asjof/ojac008 PMC9113840 35592181

[4] Pagani Bagliacca E , Ryder TJ, Lanfranchi L. Body perception and motivations in patients undergoing cryolipolysis: results from a patient questionnaire. Dermatol Surg. 2021;47(7):1018–1020. doi: 10.1097/DSS.0000000000002786 33625147

[5] Gueta R , Blaugrund E, Bloomenfeld A, Herbst KL. RZL-012, a new fat dissolving molecule, tested in Dercum’s disease patients. Dermatol Surg. 2021;47(8):1165–1166. doi: 10.1097/DSS.0000000000002989 33867475

[6] Shridharani SM , Dayan S, Biesman B, et al Efficacy and safety of tapencarium (RZL-012) in submental fat reduction. Aesthet Surg J. 2023;43(10):NP797–NP806. doi: 10.1093/asj/sjad195 37348516 PMC10501747

[7] Blaugrund E , Gueta R, Zernov A, Bloomenfeld A. Mode of action of RZL-012, a new fat-reducing molecule. Dermatol Surg. 2021;47(12):1601–1605. doi: 10.1097/DSS.0000000000003245 34537791 PMC8612913

[8] Atiyeh BS , Fadul R Jr, Chahine F. Cryolipolysis (CLL) for reduction of localized subcutaneous fat: review of the literature and an evidence-based analysis. Aesthetic Plast Surg. 2020;44(6):2163–2172. doi: 10.1007/s00266-020-01869-x 32696167

[9] Duncan DI . Safety and efficacy of simultaneous application of high-intensity focused electromagnetic field and synchronized radiofrequency for non-invasive fat reduction and muscle toning in inner thighs: magnetic resonance imaging evaluation. J Clin Aesthet Dermatol. 2022;15(8):28–32. 36061479 PMC9436229

[10] Jacob C , Kent D, Ibrahim O. Efficacy and safety of simultaneous application of HIFEM and synchronized radiofrequency for abdominal fat reduction and muscle toning: a multicenter magnetic resonance imaging evaluation study. Dermatol Surg. 2021;47(7):969–973. doi: 10.1097/DSS.0000000000003086 34001694

[11] Katz B . Concomitant use of radiofrequency and high intensity focused electromagnetic field energies for full-body remodeling: MRI evidence-based prefatory trial. J Cosmet Dermatol. 2023;22(1):193–199. doi: 10.1111/jocd.14736 36045514 PMC10087156

[12] Kent DE , Jacob C, Kinney BM. Retrospective analysis of high-intensity focused electromagnetic procedure synchronized with radiofrequency energy for visceral fat reduction. J Cosmet Dermatol. 2023;22(9):2485–2491. doi: 10.1111/jocd.15784 37154787

[13] Rambhia PH , Turner L, Ugonabo N, Chapas A. Muscle stimulation for aesthetic body shaping: a comprehensive and critical review. Dermatol Surg. 2022;48(10):1076–1082. doi: 10.1097/DSS.0000000000003550 35985005

[14] Cunha KS , Lima F, Cardoso RM. Efficacy and safety of injectable deoxycholic acid for submental fat reduction: a systematic review and meta-analysis of randomized controlled trials. Expert Rev Clin Pharmacol. 2021;14(3):383–397. doi: 10.1080/17512433.2021.1884070 33523775

[15] Anvery N , Wan HT, Dirr MA, et al Utility of high-resolution ultrasound in measuring subcutaneous fat thickness. Lasers Surg Med. 2022;54(9):1189–1197. doi: 10.1002/lsm.23604 36183386

[16] Juhasz M , Leproux A, Durkin A, Tromberg B, Mesinkovska NA. Use of a novel, noninvasive imaging system to characterize metabolic changes in subcutaneous adipose tissue after cryolipolysis. Dermatol Surg. 2020;46(11):1461–1464. doi: 10.1097/DSS.0000000000002135 31567606

